# In Vitro Characterization of Multidrug-Resistant Influenza A(H1N1)pdm09 Viruses Carrying a Dual Neuraminidase Mutation Isolated from Immunocompromised Patients

**DOI:** 10.3390/pathogens9090725

**Published:** 2020-09-02

**Authors:** Emi Takashita, Seiichiro Fujisaki, Masaru Yokoyama, Masayuki Shirakura, Hiroko Morita, Kazuya Nakamura, Noriko Kishida, Tomoko Kuwahara, Hironori Sato, Ikuko Doi, Yuji Sato, Shinichi Takao, Yukie Shimazu, Takeshi Shimomura, Takuo Ito, Shinji Watanabe, Takato Odagiri

**Affiliations:** 1 Influenza Virus Research Center, National Institute of Infectious Diseases, Tokyo 208-0011, Japan; seifuji@nih.go.jp (S.F.); masas@nih.go.jp (M.S.); h-morita@nih.go.jp (H.M.); kazuyan@nih.go.jp (K.N.); kishidan@nih.go.jp (N.K.); kuwahara@nih.go.jp (T.K.); sw@nih.go.jp (S.W.); todagiri@nih.go.jp (T.O.); 2 Pathogen Genomics Center, National Institute of Infectious Diseases, Tokyo 208-0011, Japan; yokoyama@nih.go.jp (M.Y.); hirosato@nih.go.jp (H.S.); 3 Ibaraki Prefectural Institute of Public Health, Ibaraki 310-0852, Japan; i.doi@pref.ibaraki.lg.jp; 4 Tsukuba Memorial Hospital, Ibaraki 300-2622, Japan; satou_yuji@tsukuba-kinen.or.jp; 5 Hiroshima Prefectural Technology Research Institute, Hiroshima 734-0007, Japan; s-takaoe1077@pref.hiroshima.lg.jp (S.T.); y-shimazu89236@pref.hiroshima.lg.jp (Y.S.); 6 National Hospital Organization Hiroshimanishi Medical Center, Hiroshima 739-0696, Japan; shimomuratakeshi2010@gmail.com; 7 National Hospital Organization Kure Medical Center, Hiroshima 737-0023, Japan; itot@kure-nh.go.jp

**Keywords:** influenza, neuraminidase inhibitor, oseltamivir, peramivir, zanamivir, laninamivir, baloxavir, favipiravir, resistance

## Abstract

Influenza A(H1N1)pdm09 viruses carrying a dual neuraminidase (NA) substitution were isolated from immunocompromised patients after administration of one or more NA inhibitors. These mutant viruses possessed an H275Y/I223R, H275Y/I223K, or H275Y/G147R substitution in their NA and showed enhanced cross-resistance to oseltamivir and peramivir and reduced susceptibility to zanamivir compared to single H275Y mutant viruses. Baloxavir could be a treatment option against the multidrug-resistant viruses because these dual H275Y mutant viruses showed susceptibility to this drug. The G147R substitution appears to stabilize the NA structure, with the fitness of the H275Y/G147R mutant virus being similar or somewhat better than that of the wild-type virus. Since the multidrug-resistant viruses may be able to transmit between humans, surveillance of these viruses must continue to improve clinical management and to protect public health.

## 1. Introduction

In Japan, four neuraminidase (NA) inhibitors––oseltamivir, peramivir, zanamivir, and laninamivir––and a cap-dependent endonuclease inhibitor, baloxavir marboxil, have been approved for the treatment of influenza [1]. In addition, favipiravir, a viral RNA-dependent RNA polymerase inhibitor, has been approved for influenza pandemic preparedness [2]. Since nationwide monitoring is important for public health planning and clinical management, we have been conducting surveillance of antiviral-resistant viruses.

During our surveillance, we detected multidrug-resistant influenza A(H1N1)pdm09 viruses from immunocompromised patients [3,4,5]. These viruses possessed an I223K, an I223R, or a G147R substitution in combination with an H275Y substitution in their NA protein. Several studies have reported the detection of A(H1N1)pdm09 viruses carrying a dual H275Y substitution, such as H275Y/E119D, H275Y/E119G, H275Y/I223K, H275Y/I223R, and H275Y/S247N, from immunocompromised patients [6,7,8,9,10,11]. A few studies have been carried out to understand the impact of the H275Y/E119D and H275Y/I223R substitutions on viral fitness [12,13,14]; however, that of the H275Y/I223K and H275Y/G147R substitutions remains unknown. Here, we report our assessment of the in vitro properties of H275Y/I223K, H275Y/I223R, and H275Y/G147R dual mutant viruses isolated from immunocompromised patients.

## 2. Materials and Methods

### 2.1. Viruses

The dual H275Y mutant viruses (H275Y/I223K, H275Y/I223R, and H275Y/G147R) were detected from immunocompromised patients hospitalized in hematology departments after administration of one or more NA inhibitors (Table 1). The representative single H275Y mutant and wild-type viruses detected during the same influenza season are shown in Table 2. These representative viruses are genetically most closely related to the corresponding dual H275Y mutant viruses in the Global Initiative on Sharing All Influenza Data (GISAID) EpiFlu database (http://www.gisaid.org). A/Hiroshima/57/2014, A/Osaka/8/2014, and A/Sakai/23/2013 have hemagglutinin (HA) genes that belong to genetic clade 6B. A/Ibaraki/54/2016, A/Yokohama/94/2016, and A/Yokohama/40/2016 have HA genes that belong to genetic clade 6B.1. A/Hiroshima/13/2016, A/Aichi/83/2016, and A/Yokohama/59/2016 have HA genes that belong to genetic clade 6B.2. All viruses were propagated in MDCK (NBL-2) cells (ATCC CCL-34). The dual substitutions were confirmed in both clinical specimens and isolates by sequencing them.

### 2.2. Antiviral Compounds

Oseltamivir acid, peramivir trihydrate, and zanamivir hydrate were purchased from Biosynth Carbosynth (Berkshire, UK). Laninamivir was provided by Daiichi Sankyo (Tokyo, Japan). Baloxavir acid was purchased from MedChemExpress (Monmouth Junction, NJ, USA), and favipiravir was purchased from Cayman Chemical (Ann Arbor, MI, USA). Oseltamivir, peramivir, zanamivir, laninamivir, and favipiravir were dissolved in distilled water, and baloxavir was dissolved in dimethyl sulfoxide.

### 2.3. NA Inhibition Assay

NA inhibitor susceptibilities were determined by using a fluorescence-based NA inhibition assay with the NA-Fluor influenza neuraminidase assay kit (Applied Biosystems, Foster City, CA, USA) and 4-MU-NANA substrate (Biosynth Carbosynth). Briefly, diluted viruses were mixed with 20-fold serial dilutions of 125,000 nM oseltamivir, peramivir, zanamivir, or laninamivir and incubated for 20 min at 37 °C; 4-MU-NANA substrate was then added, and the mixture was incubated for 30 min at 37 °C. The reaction was stopped by adding 0.12 M Na_2_CO_3_ in 40% ethanol. The fluorescence of the solution was measured at an excitation wavelength of 355 nm and an emission wavelength of 460 nm. The results are expressed as 50% inhibitory concentration (IC_50_) values, which were calculated by using GraphPad Prism (GraphPad Software, San Diego, CA, USA).

### 2.4. Focus Reduction Assay

Baloxavir susceptibilities were determined by using a focus reduction assay as previously described [1] in humanized MDCK cells (i.e., hCK cells), which express high levels of α2,6-sialoglycans and very low levels of α2,3-sialoglycans [15]. hCK cells were kindly provided by Dr. Yoshihiro Kawaoka (University of Wisconsin–Madison). hCK cells in 96-well plates were infected with 1000 focus-forming units (FFU)/well of viruses. Virus adsorption was carried out for 1 h at 37 °C and then an equal volume of 1.2% Avicel RC-581 (DuPont Nutrition USA, Wilmington, DE, USA) in culture medium containing serial dilutions (0.025–2500 nM) of baloxavir was added to each well in triplicate. The cells were incubated for 24 h at 34 °C and then fixed with formalin. After the formalin was removed, the cells were immunostained with a mouse monoclonal antibody against influenza A virus nucleoprotein (Merck KGaA, Darmstadt, Germany), followed by a horseradish peroxidase-labeled goat anti-mouse immunoglobulin (SeraCare Life Sciences, Milford, MA, USA). The infected cells were stained with TrueBlue Substrate (SeraCare Life Sciences) and then washed with distilled water. After cell drying, the focus numbers were quantified by using an ImmunoSpot S6 Analyzer, ImmunoCapture software, and BioSpot software (Cellular Technology, Cleveland, OH, USA). The results are expressed as IC_50_ values.

### 2.5. Cytopathic Effect Reduction Assay

Favipiravir susceptibilities were determined by using a cytopathic effect reduction assay as previously described [2] in AX4 cells, which overexpress α2,6-sialoglycans [16]. AX4 cells were kindly provided by Dr. Yoshihiro Kawaoka. AX4 cells in 96-well plates were infected with viruses at a multiplicity of infection (MOI) of 0.01 50% tissue culture infective dose (TCID_50_)/cell. Virus adsorption was carried out for 1 h at 37 °C and then an equal volume of culture medium containing serial dilutions (0.05–1000 nM) of favipiravir was added to each well in triplicate. The cells were incubated for 3–5 days at 34 °C. To determine the extent of the cytopathic effect, the CellTiter-Glo 2.0 Assay reagent (Promega Corporation, Madison, WI, USA) was added and luminescence was measured. The results are expressed as 50% effective concentration (EC_50_) values, which were calculated by using GraphPad Prism.

### 2.6. Plaque Assay

Virus titrations were conducted by using a plaque assay as previously described [2] in AX4 cells. AX4 cells in 6-well plates were infected with serial dilutions of viruses in triplicate. Virus adsorption was carried out for 1 h at 37 °C. After the inoculum was removed, 0.8% agarose in culture medium was added to each well. The cells were incubated for 3 days at 34°C and the plaque numbers were counted.

### 2.7. Virus Replication Kinetics In Vitro

In vitro replication kinetics of the dual H275Y mutant viruses were determined as previously described [17] in AX4 cells. AX4 cells were infected with viruses at an MOI of 0.001 plaque-forming units (PFU)/cell in triplicate. The cells were incubated at 34°C. The supernatants were harvested at 12, 24, 36, 48, 60, and 72 h post-infection and were subjected to virus titration by using plaque assays.

### 2.8. Competitive Virus Replication In Vitro

The competitive growth capability of each dual H275Y mutant virus with that of the wild-type virus was compared as previously described [18] in AX4 cells. Each dual H275Y mutant virus was coinfected with the corresponding wild-type virus at an MOI of 0.01 PFU/cell in triplicate. The cells were incubated at 34 °C. At 2 days post-infection, the supernatants were subjected to virus titration by using plaque assays and to deep sequencing analysis to determine the relative proportion of each genotype. The viruses were serially passaged 3–4 times at an MOI of 0.01 PFU/cell.

### 2.9. Deep Sequencing Analysis

Deep sequencing analysis was performed as previously described [2]. A cDNA library was prepared from viral RNA by using the NEBNext Ultra RNA Library Prep Kit for Illumina and NEBNext Singleplex Oligos for Illumina (New England Biolabs, Ipswich, MA, USA), followed by purification by using Agencourt AMPure XP (Beckman Coulter, Brea, CA, USA). The library was sequenced by using MiSeq Reagent Kits v2 with MiSeq (Illumina, San Diego, CA, USA). Sequence reads were aligned to the reference sequence of A/California/07/2009(H1N1)pdm09 by using CLC Genomics Workbench 8 (CLC bio, Aarhus, Denmark).

### 2.10. Structural Analysis of the NA Protein

A structure model of the NA protein of each mutant virus was constructed by use of homology modeling and was refined by using Molecular Operating Environment (MOE) (Chemical Computing Group, Montreal, Canada) as previously described [18]. The crystal structure of the A(H1N1)pdm09 virus NA protein (PDB ID 4B7R; resolution, 1.9 Å) [19] served as the modeling template. Single-point mutations were generated on the NA model. Ensembles of the protein conformations were generated by using the LowMode MD module in MOE. The average stability changes of the ensembles were calculated by using the Boltzmann distribution. The stability scores (∆∆Gs) of the structures were obtained through the stability scoring function of the Protein Design application.

### 2.11. Statistical Analysis

Statistical analyses were performed using GraphPad Prism. Statistically significant differences between groups were determined by using an unpaired t-test calculated by fitting a mixed-effects model. *p* values of <0.05 were considered statistically significant.

## 3. Results

### 3.1. Immunocompromised Patients Infected with Dual H275Y Mutant Viruses

The clinical courses of the immunocompromised patients infected with the dual H275Y mutant viruses are shown in Table 1. The first patient, a woman in her late 70s who was infected with the H275Y/I223R mutant virus (A/Hiroshima/57/2014), was treated with peramivir (600 mg) on the day of symptom onset. After four days of peramivir treatment, the patient’s symptoms had not improved, and she was therefore treated with laninamivir (20 mg). She recovered after laninamivir administration. The second patient, a man in his late 70s who was infected with the H275Y/I223K mutant virus (A/Ibaraki/54/2016), was treated with peramivir (300 mg) on the day of symptom onset. His infection persisted for more than two weeks, so he then received combination therapy of peramivir (300 mg) and oseltamivir (75 mg twice daily) for seven days. However, the influenza rapid diagnostic test remained positive. Laninamivir (40 mg) was administered two days after the end of the combination therapy and after six days of laninamivir administration, the rapid diagnostic test was negative. The third patient, a woman in her early 50s who was infected with the H275Y/G147R mutant virus (A/Hiroshima/13/2016), received prophylaxis with laninamivir (40 mg). Three days later, she had onset of illness and was treated with peramivir (600 mg) for five days. Her infection persisted for more than one month and peramivir (600 mg) was administered for three intermittent periods of five days. She developed left lower lobe pneumonia 20 days post-disease onset and alveolar hemorrhage 12 days after. Two days after the end of the third five-day course, she died.

### 3.2. Antiviral Susceptibilities of the Dual H275Y Mutant Viruses

We compared the susceptibility of the dual H275Y mutant viruses (A/Hiroshima/57/2014, A/Ibaraki/54/2016, and A/Hiroshima/13/2016) with that of the corresponding single H275Y mutant (A/Osaka/8/2014, A/Yokohama/94/2016, and A/Aichi/83/2016) and wild-type (A/Sakai/23/2013, A/Yokohama/40/2016, and A/Yokohama/59/2016) viruses to four NA inhibitors (oseltamivir, peramivir, zanamivir, and laninamivir), a cap-dependent endonuclease inhibitor, baloxavir, and a viral RNA-dependent RNA polymerase inhibitor, favipiravir (Table 2). The dual H275Y mutant viruses are genetically most closely related to the corresponding single H275Y mutant and wild-type viruses. The H275Y/I223R, H275Y/I223K, and H275Y/G147R mutant viruses exhibited enhanced cross-resistance to oseltamivir and peramivir and reduced susceptibility to zanamivir compared to the corresponding single H275Y mutant viruses. The H275Y/I223R and H275Y/I223K mutant viruses, but not the H275Y/G147R mutant virus, showed reduced susceptibility to laninamivir. All viruses tested were susceptible to baloxavir and favipiravir.

### 3.3. In Vitro Replication Kinetics of the Dual H275Y Mutant Viruses

**Figure 1:**
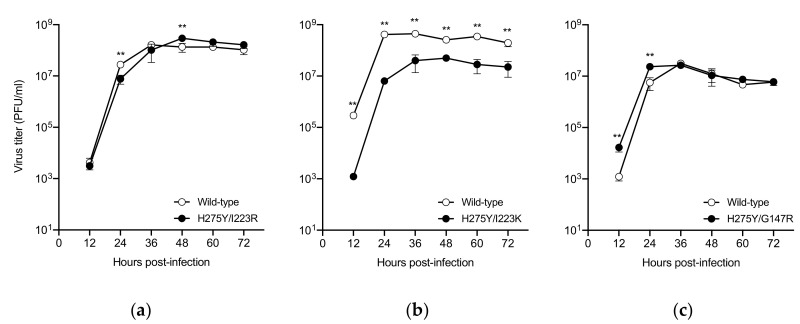
In vitro replication kinetics of the dual H275Y mutant influenza A(H1N1)pdm09 viruses. AX4 cells were infected with the H275Y/I223R, H275Y/I223K, or H275Y/G147R mutant virus, or the corresponding wild-type virus at a multiplicity of infection of 0.001 plaque-forming units (PFU)/cell in triplicate. The supernatants were harvested at the indicated time and were subjected to virus titration by using plaque assays. Means and standard deviations are shown. Asterisks indicate statistically significant differences between the dual H275Y mutant and wild-type viruses as determined by using unpaired t-test calculated by fitting a mixed-effects model; ** *p* < 0.01. Wild-type: (**a**) A/Sakai/23/2013, (**b**) A/Yokohama/40/2016, and (**c**) A/Yokohama/59/2016.

The impact of the dual substitution on viral growth was assessed in AX4 cells, which overexpress the human influenza receptor (α2,6-sialoglycans) (Figure 1). Since each of the wild-type viruses has different genetic backgrounds, the virus titers of the wild-type viruses cannot be compared directly with each other. The virus titers of the H275Y/I223R mutant virus were found to be comparable to those of the corresponding wild-type virus as previously described [13]. The replication of the H275Y/I223K mutant virus was significantly reduced compared to that of the corresponding wild-type virus. The H275Y/G147R mutant and the corresponding wild-type viruses had comparable virus titers after 36 h post-infection; however, the dual mutant virus replicated more efficiently than the wild-type virus during the initial cycle of infection. The G147R substitution confers receptor-binding activity to the NA protein of the A(H1N1)pdm09 virus [20]. A higher receptor-binding activity may affect faster virus replication at the early stage of infection. These results indicate that the H275Y/I223K substitution negatively affects viral growth, at least in vitro, but that the H275Y/I223R and H275Y/G147R substitutions do not.

### 3.4. Competitive Growth Capabilities of the Dual H275Y Mutant Viruses and the Wild-Type Viruses

**Figure 2:**
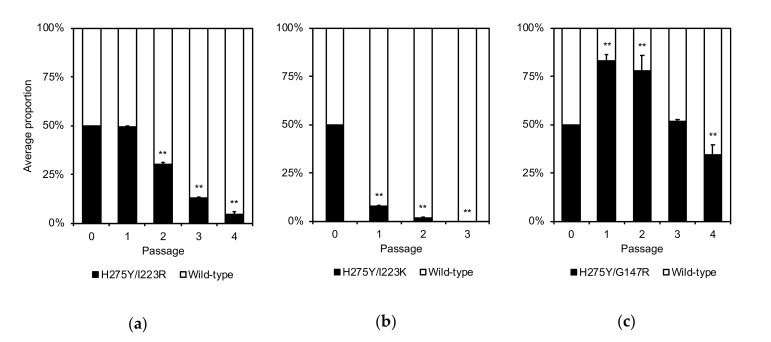
Competitive growth capabilities of the dual H275Y mutant influenza A(H1N1)pdm09 viruses and the wild-type viruses. AX4 cells were coinfected with the H275Y/I223R, H275Y/I223K, or H275Y/G147R mutant virus and with the corresponding wild-type virus at a multiplicity of infection (MOI) of 0.01 plaque-forming units (PFU)/cell in triplicate. At two days post-infection, the supernatants were subjected to virus titration by using plaque assays and to deep sequencing analysis to determine the relative proportion of each genotype. The viruses were serially passaged 3–4 times at an MOI of 0.01 PFU/cell. Means and standard deviations are shown. Asterisks indicate statistically significant differences between the dual H275Y mutant and wild-type viruses as determined by using unpaired t-test calculated by fitting a mixed-effects model; ** *p* < 0.01. Wild-type: (**a**) A/Sakai/23/2013, (**b**) A/Yokohama/40/2016, and (**c**) A/Yokohama/59/2016.

To compare the competitive growth capability of the dual H275Y mutant viruses with that of wild-type viruses, each dual H275Y mutant virus was coinfected with its corresponding wild-type counterpart (Figure 2). The proportion of the H275Y/I223R mutant virus to that of the wild-type virus was similar at passage 1 and then decreased significantly, whereas the proportion of the H275Y/I223K mutant virus to that of the wild-type virus decreased significantly from passage 1. In contrast, the H275Y/G147R mutant virus rapidly became dominant in the mixed virus populations at passages 1 and 2, indicating that this mutant virus continued to replicate efficiently, competing with the wild-type virus during the initial cycle of infection. These results indicate that the growth capability of the H275Y/G147R virus is comparable to, or somewhat better than, that of the wild-type virus at least in vitro.

### 3.5. Effects of Amino Acid Substitutions on the Stability of the NA Protein

**Figure 3:**
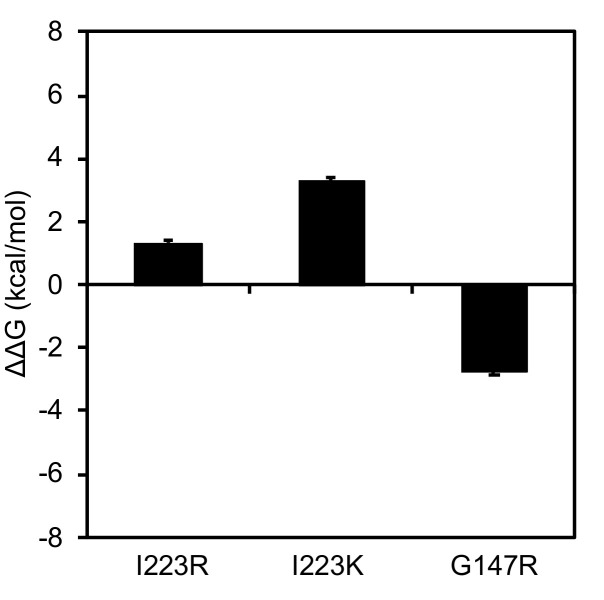
Structural analysis of the influenza A(H1N1)pdm09 virus neuraminidase (NA) protein. Structure models of the NA proteins were constructed by use of homology modeling. Single-point mutations were generated on the NA model. The average stability changes were calculated by using the Boltzmann distribution. Means and standard deviations of the stability scores (∆∆Gs) of the structures are shown.

The H275Y substitution caused a detrimental effect on viral fitness by decreasing the stability of the NA protein [21]. Additional substitutions, V241I and N369K, were thought to improve the stability of the NA, thereby compensating for the negative effects of the H275Y substitution on the growth and transmissibility of the mutant virus [22,23]. To assess the effect of the I223R, I223K, and G147R substitutions on the stability of the NA protein, we performed an in silico mutagenesis study as previously described [18] (Figure 3). The changes in stability caused by each of the substitutions I223R, I223K, and G147R were 1.32, 3.29, and -2.72 kcal/mol, respectively. These data suggest that the I223R and I223K substitutions may destabilize the NA structure, whereas the G147R substitution likely stabilizes NA, consistent with our previous finding [4].

## 4. Discussion

Immunocompromised patients are at great risk for emergence of NA inhibitor-resistant viruses [24,25,26,27]. The high frequency of resistant viruses among immunocompromised patients is associated with the high levels of viral titers and prolonged viral shedding in these patients [28,29,30,31]. A(H1N1)pdm09 viruses carrying the single H275Y substitution rapidly emerge during treatment with oseltamivir and/or peramivir in immunocompromised patients [32,33] and exhibit clinically significant cross-resistance to oseltamivir and peramivir [34]. In the present study, the dual H275Y mutant A(H1N1)pdm09 viruses showed enhanced cross-resistance to oseltamivir and peramivir and reduced susceptibility to zanamivir compared to the single H275Y mutant viruses. Patients infected with the H275Y/I223R or H275Y/I223K mutant virus showed improved clinical and virologic responses after laninamivir treatment, although the laninamivir susceptibilities of these viruses were reduced. The H275Y/G147R mutant virus retained susceptibility to laninamivir, but the patient infected with this mutant virus did not have the opportunity to receive laninamivir treatment before she died. All of the dual H275Y mutant viruses tested were susceptible to baloxavir and favipiravir. Baloxavir was approved for the treatment of influenza A and B virus infections in February 2018 in Japan; favipiravir has been approved and stockpiled for use against novel influenza virus infections for which first-line antivirals are ineffective [1]. Therefore, baloxavir could be a treatment option against these multidrug-resistant viruses.

The selective pressure from prolonged exposure to NA inhibitors in immunocompromised patients can lead to the emergence of multidrug resistance. The patients infected with the H275Y/I223K or H275Y/G147R mutant virus received prolonged treatment with oseltamivir and/or peramivir, whereas the patient infected with the H275Y/I223R mutant virus was treated with a single dose of peramivir before specimen collection. A pretreatment specimen from this patient was unavailable, but the specimen collected after four days of peramivir treatment contained the dual H275Y/I223R substitution and not a mixture including wild-type 275H and 223I. These observations suggest three possibilities: the patient was infected by another host harboring the single H275Y mutant virus, the patient was infected by another host harboring the single I223R mutant virus, or the patient was infected by another host harboring the dual H275Y/I223R mutant virus. Since the NA inhibitor-resistant viruses transmit well in the immunocompromised patients [35,36], we cannot rule out human-to-human transmission of these mutant viruses.

The growth capability of the H275Y/I223K mutant virus was significantly reduced compared to that of the wild-type virus, and the H275Y/I223R mutant virus retained growth capability to some extent. Residue 223 is located within the framework of the NA enzymatic active site [37]. Our structural analysis predicted that the I223K and I223R substitutions destabilize the NA structure, but the stability score for I223K was higher than that for I223R. These results suggest that an NA with the I223K substitution is less stable than an NA with the I223R substitution. Therefore, the H275Y/I223K mutant virus showed reduced viral fitness relative to the H275Y/I223R mutant virus.

A large cluster of the H275Y mutant A(H1N1)pdm09 virus occurred in Sapporo, Japan during the 2013–14 season [18]. This mutant virus emerged prior to the main influenza season and spread predominantly in the community. To understand the reason for this large cluster, we examined the in vitro and in vivo properties of the mutant virus. We found that it grew well in vitro and in vivo, with growth similar to, or somewhat better than, the wild-type virus. However, the mutant NA structure was less stable than that of the wild-type virus. Therefore, once the wild-type virus began to circulate in the community, the mutant virus could not compete and faded out. In the present study, we found that the H275Y/G147R mutant virus retained the ability to grow similarly to, or somewhat better than, the wild-type virus. Residue 147 is located in a 150-loop adjacent to the NA enzymatic active site [4,5]. In our structural analysis, the G147R substitution was predicted to stabilize the NA structure. Therefore, the H275Y/G147R mutant virus retained its viral fitness and might be as transmissible among humans as the wild-type virus. In fact, the patient infected with this mutant virus developed pneumonia without the isolation of bacterial pathogens, suggesting viral pneumonia with this mutant virus [4]. The in vivo properties of this mutant virus should be further investigated.

The H275Y substitution in the A(H1N1)pdm09 virus NA protein compromises viral fitness; however, additional V241I and N369K substitutions in the NA protein were reported to increase the replication and transmission fitness of the H275Y mutant A(H1N1)pdm09 viruses [22,23]. The dual H275Y mutant viruses in the present study possess these permissive substitutions. Furthermore, currently circulating A(H1N1)pdm09 viruses also possess these substitutions, suggesting an increased risk for oseltamivir and peramivir cross-resistant viruses to emerge and spread. The emergence of the multidrug-resistant viruses reduces the antiviral options for treatment. This report highlights the importance of closely monitoring multidrug-resistant viruses in immunocompromised patients to improve clinical management and surveillance of these resistant viruses to protect public health.

## Figures and Tables

**Table 1:** Immunocompromised patients infected with the dual H275Y mutant influenza A(H1N1)pdm09 viruses.

Isolate Name	Date of Symptom Onset (Day/Month/Year)	Antiviral Administration (Day/Month/Year)	Date of Specimen Collection (Day/Month/Year)	NA Substitution
A/Hiroshima/57/2014	31-03-2014	31-03-2014: Peramivir 04-04-2014: Laninamivir	04-04-2014	H275Y/I223R
A/Ibaraki/54/2016	09-02-2016	09-02-2016: Peramivir 24-02-2016 to 03-03-2016: Peramivir 26-02-2016 to 03-03-2016: Oseltamivir 05-03-2016: Laninamivir	07-03-2016	H275Y/I223K
A/Hiroshima/13/2016	26-02-2016	23-02-2016: Laninamivir 26-02-2016 to 01-03-2016: Peramivir 07-03-2016 to 11-03-2016: Peramivir 28-03-2016 to 01-04-2016: Peramivir	11-03-2016	H275Y/G147R

NA: neuraminidase.

**Table 2:** Antiviral susceptibilities of the dual H275Y mutant influenza A(H1N1)pdm09 viruses.

Isolate Name	GISAID Isolate ID	NA Substitution	IC_50_, nM (Fold-Change ^1^)					EC_50_, µM (Fold-Change ^1^)
			Oseltamivir ^2^	Peramivir ^2^	Zanamivir ^2^	Laninamivir ^2^	Baloxavir ^3^	Favipiravir ^4^
A/Hiroshima/57/2014	EPI ISL 160499	H275Y/I223R	6263.69 (20,000)	944.20 (94,000)	4.84 (48)	4.48 (20)	8.34 (2.1)	7.99 (1.2)
A/Osaka/8/2014	EPI ISL 155839	H275Y	173.80 (560)	28.25 (2,800)	0.03 (0.3)	0.14 (0.6)	5.10 (1.3)	4.76 (0.7)
A/Sakai/23/2013	EPI ISL 154461	None (wild-type)	0.31	0.01	0.10	0.22	4.05	6.64
A/Ibaraki/54/2016	EPI ISL 221789	H275Y/I223K	10161.87 (16,000)	501.62 (7,200)	2.48 (12)	1.94 (6.9)	7.24 (1.2)	10.20 (1.0)
A/Yokohama/94/2016	EPI ISL 218900	H275Y	466.79 (730)	20.88 (300)	0.33 (1.6)	0.52 (1.9)	3.49 (0.6)	21.40 (2.1)
A/Yokohama/40/2016	EPI ISL 217919	None (wild-type)	0.64	0.07	0.21	0.28	6.16	10.01
A/Hiroshima/13/2016	EPI ISL 220376	H275Y/G147R	1324.62 (1,500)	114.14 (1,400)	1.56 (4.5)	0.37 (0.9)	10.59 (1.0)	15.13 (1.7)
A/Aichi/83/2016	EPI ISL 233222	H275Y	364.44 (420)	31.93 (400)	0.77 (2.2)	0.77 (1.9)	10.18 (0.9)	14.83 (1.7)
A/Yokohama/59/2016	EPI ISL 217920	None (wild-type)	0.86	0.08	0.35	0.40	11.04	8.81

GISAID: Global Initiative on Sharing All Influenza Data; NA: neuraminidase; IC_50_: 50% inhibitory concentration; EC_50_: 50% effective concentration. ^1^ Fold-change in IC_50_ or EC_50_ values compared with the wild-type viruses. ^2^ IC_50_ values were determined by using an NA inhibition assay. ^3^ IC_50_ values were determined by using a focus reduction assay. ^4^ EC_50_ values were determined by using a cytopathic effect reduction assay.
